# HIRA dependent H3.3 deposition is required for transcriptional reprogramming following nuclear transfer to *Xenopus* oocytes

**DOI:** 10.1186/1756-8935-5-17

**Published:** 2012-10-29

**Authors:** Jerome Jullien, Carolina Astrand, Emmanuelle Szenker, Nigel Garrett, Genevieve Almouzni, John B Gurdon

**Affiliations:** 1 The Welcome Trust/Cancer Research UK Gurdon Institute, The Henry Welcome building of Cancer and Developmental Biology, and Department of Zoology, Cambridge, University of Cambridge, Tennis Court Road, Cambridge, CB2 1QN, UK; 2 Institute Curie Section de Recherché UMR 218, 26 rue d'Ulm, 75248, Paris Cedex 05, France

## Abstract

**Background:**

Nuclear reprogramming is potentially important as a route to cell replacement and drug discovery, but little is known about its mechanism. Nuclear transfer to eggs and oocytes attempts to identify the mechanism of this direct route towards reprogramming by natural components. Here we analyze how the reprogramming of nuclei transplanted to *Xenopus* oocytes exploits the incorporation of the histone variant H3.3.

**Results:**

After nuclear transplantation, oocyte-derived H3.3 but not H3.2, is deposited on several regions of the genome including rDNA, major satellite repeats, and the regulatory regions of Oct4. This major H3.3 deposition occurs in absence of DNA replication, and is HIRA-and transcription-dependent. It is necessary for the shift from a somatic- to an oocyte-type of transcription after nuclear transfer.

**Conclusions:**

This study demonstrates that the incorporation of histone H3.3 is an early and necessary step in the direct reprogramming of somatic cell nuclei by oocyte. It suggests that the incorporation of histone H3.3 is necessary during global changes in transcription that accompany changes in cell fate.

## Background

Nuclear reprogramming is characterized by a global shift in gene expression. The mechanisms underlying this phenomenon are not well understood but are likely to involve changes to chromatin. For example, an increase in histone H3K4 methylation has been observed in nuclei following nuclear transfer (NT) and during iPS production [1,2]. Alternatively, the incorporation of histone variants into chromatin can provide another way to drastically alter the structure of chromatin. Nucleosomes containing core histone variants H3.3 or macroH2A have been associated with the active and inactive states of a gene, respectively. MacroH2A restricts the reactivation of pluripotency genes from mouse nuclei transplanted to *Xenopus* oocytes [3]. In nuclear transfer to *Xenopus* eggs, histone H3.3 participates in the transmission of an active state of a gene, even in embryonic lineages where genes should be silenced [4]. Furthermore, histone variants are also positively involved in the mechanism of transcriptional reprogramming. We have previously shown that the incorporation of histone B4, an oocyte specific linker histone variant, is a necessary step for nuclear reprogramming following nuclear transfer [5]. A number of histone changes are already known to be associated with nuclear reprogramming by eggs and oocytes. While those observed in eggs may well be related to DNA synthesis and cell replication coupled events during the cell cycle, those that take place in somatic nuclei transplanted to oocytes which do not replicate DNA and are arrested in prophase I of meiosis are associated essentially with new transcription and are independent of cell cycle progression.

Here we investigate the dynamics of histone H3 variants in the reprogramming of mammalian nuclei transplanted to *Xenopus* oocytes. In this type of reprogramming there is no cell division and new cell types are not derived. However, the transplanted nuclei undergo dramatic changes in their pattern of gene expression so that transcription is switched directly from a somatic to an oocyte type. The evolutionarily conserved histone variant H3.3 has been found to be especially enriched in the coding region of transcriptionally active genes as well as in gene regulatory elements [6]. This histone is often associated with histone modifications related to gene activation [7,8]. Histone H3.3 can be incorporated into chromatin throughout the cell cycle in a replication independent manner by the histone chaperone HIRA [9,10], which is also found to be required for global H3.3 deposition in the male pronucleus after fertilization in Drosophila [11]. This association between histone H3.3 and the HIRA deposition pathway has been further demonstrated to play a critical role during a major change in gene expression at gastrulation in *Xenopus*[12]. Finally, early work showed that plasmid DNA injected to oocytes is differently transcribed depending on whether its chromatin has been assembled in a DNA synthesis dependent or independent manner [13]. Together these findings prompted us to investigate the importance of the histone variant H3.3 and its deposition in transcriptional reprogramming of nuclei transplanted to *Xenopus* oocytes. We demonstrate that the deposition of H3.3 by HIRA is necessary for transcriptional reprogramming. We also observe that HIRA mediated H3.3 deposition and transcription are interdependent in somatic nuclei transplanted to *Xenopus* oocytes.

## Results and discussion

### Gain and loss of histone H3 variants

**Figure 1:**
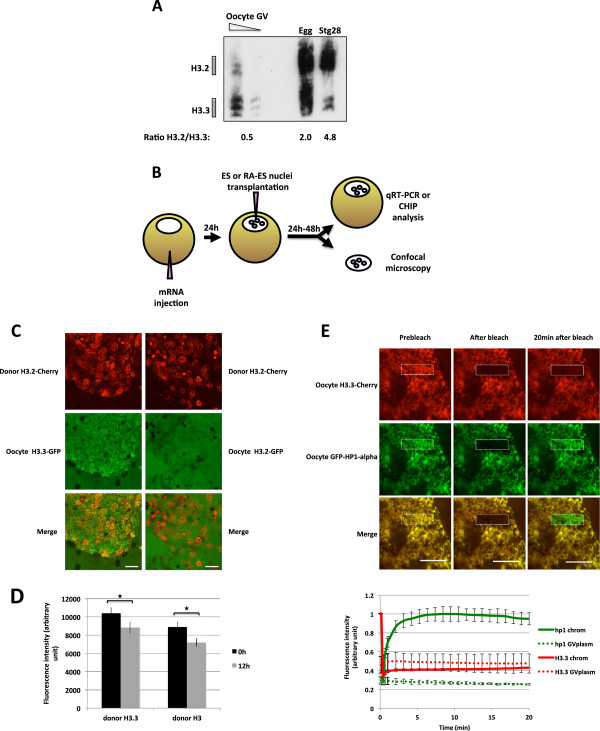
**H3.3 but not H3.2 is accumulated in transplanted nuclei.** (**A**) H3.2 *vs.* H3.3 content in oocyte GVs, eggs, and st28 embryos. Soluble pool of H3.2 and H3.3 were separated by Triton Acid Urea (TAU) gel electrophoresis and analyzed by western blot with an anti-H3 antibody. We loaded the equivalent of 20 and 10 GV, and about two eggs or st28 embryos. (**B**) Schematic outline of injection procedure, where mRNAs coding for tagged *Xenopus* histones are injected into oocyte cytoplasm 1 day before injection of donor nuclei. Oocytes containing transplanted nuclei are then incubated for 1 to 2 days and submitted to either live imaging, ChIP, or qRT-PCR analysis. (**C**) H3.2-cherry labelled ESC nuclei were transplanted to the germinal vesicle of H3.2-GFP or H3.3-GFP containing oocytes. Forty-eight hours after transplantation germinal vesicles were isolated and analyzed by confocal microscopy. Images are projection of Z-stack. (**D**) Quantification of H3.3-Cherry or H3.2-Cherry loss from transplanted nuclei. Donor ESC nuclei expressing H3.3-Cherry or H3.2-Cherry were transplanted to GVs isolated in oil and were imaged immediately (0 h) or 12 h after transplantation. The histogram shows fluorescently labeled histone signal averaged from 40 nuclei in each condition (Error bars indicate s.e.m). ★ indicate *P* value < 0.05, (T-TEST). (**E**) FRAP analysis of oocyte H3.3 and HP1alpha associated with transplanted nuclei. ESC nuclei were transplanted to H3.3-Cherry and GFP-HP1-alpha expressing oocytes. Forty-eight hours after transplantation, GVs were isolated and H3.3-cherry and GFP-HP1alpha accumulated on transplanted chromatin was subjected to FRAP analysis. Images show a clump of labeled nuclei at various time points during the FRAP procedure. The graph indicates fluorescence intensity changes over time for the tested chromatin region as well as in the surrounding GVplasm. Fluorescence intensity of H3.3 Cherry on transplanted chromatin at the beginning of the experiment is set to 1. Note that H3.3 Cherry fluorescence intensity in the GVplasm amounts to about 40% of H3.3-Cherry accumulated on chromatin, and remains constant during the course of the FRAP experiment.

In order to investigate the mechanism of transcriptional reprogramming by oocytes, we have first monitored the transfer of histone variants between the oocyte and the transplanted nuclei. We have focused our analysis on histone H3.2 and H3.3, the two non-centromeric histone H3 variants known to be present in *Xenopus*. Triton Acetic acid Urea (TAU) gel analysis indicates that in the *Xenopus* oocyte, the ratio of histone H3.3 to that of histone H3.2 is much higher than in somatic cells (Figure 1A). Indeed TAU gel analysis shows that cells of Stage 28 *Xenopus* embryo contain approximately five times less H3.3 than H3.2, whereas the *Xenopus* oocyte germinal vesicle (GV) exhibits a two-fold excess of H3.3 over H3.2. Thus, the *Xenopus* oocyte GV is characterized by an unusually high proportion of histone H3.3 variant. We have asked whether core histone variants originating from the oocyte can be deposited onto the chromatin of transplanted nuclei. For that purpose we have expressed in the oocyte, by mRNA injection, fluorescently tagged histone H3.2 and H3.3 (Figure 1B). Forty-eight hours after mRNA injection, tagged histones are expressed in the oocyte at a similar level to their endogenous counterparts (Additional file 1: Figure S1 and Figure 1A). ES nuclei expressing H3.2-cherry are then transplanted into these oocytes, and incubated for another day, when their germinal vesicles containing transplanted nuclei were isolated and analyzed by confocal microscopy (Figure 1C). H3.2-cherry marks the location of donor nuclei and 48 h after nuclear transfer, we observe an accumulation of oocyte H3.3 in transplanted chromatin an effect not seen with oocyte H3.2 (Figure 1C). At 48 h after nuclear transfer, most of the nuclei have lost nuclear membrane integrity indicating that the observed accumulation of H3.3 corresponds to its incorporation onto transplanted chromatin rather than nuclear import. When monitored by confocal analysis in real time, the incorporation of oocyte H3.3 onto transplanted chromatin is readily detectable 10 h after nuclear transfer. H3.3 incorporation steadily increases and is observed in all transplanted nuclei 15 h after nuclear transplantation (Additional file 1: Figure S2, oocyte H3.3). We conclude from these experiments that following nuclear transfer to *Xenopus* oocytes, a high incorporation of H3.3 compared to H3.2, is observed in transplanted chromatin.

We have then asked whether the loading of oocyte H3.3 onto transplanted chromatin is associated with the loss of H3.2 or H3.3 from donor nuclei. For that purpose we have used donor nuclei containing cherry labeled H3.2 or H3.3. Quantitation of the level of cherry labeled histones that remain associated with donor nuclei after transplantation to oocytes was done by confocal microscopy. We observed that during the first 12 h after nuclear transfer 15% and 19% of H3.2-cherry or H3.3-cherry, respectively, are lost from transplanted nuclei (Figure 1D). This suggests that following nuclear transfer to *Xenopus* oocytes, a significant fraction of H3.2 and H3.3 in donor nuclei is replaced by oocyte H3.3. The mobility of core histones on the chromatin of nuclei transplanted to *Xenopus* oocytes is not known. Changes in histone mobility have been proposed to account for plasticity in transcription [14] and could therefore account for the change in gene expression observed after nuclear transfer. We therefore investigated whether histone H3.3 incorporated after transplantation corresponds to a fraction of the transplanted nuclear core histone that has become highly mobile. When tested by FRAP analysis over a period of 20 min, we observed that oocyte derived H3.3 loaded onto transplanted chromatin does not recover above the level of H3.3 found in the surrounding germinal vesicle (Figure 1E). By contrast oocyte derived HP1-alpha fully recovers within the 20 min following photo bleaching, indicating that within the imaging conditions used some chromatin associated protein exhibits rapid turnover. We therefore conclude that, once incorporated into transplanted nuclei, the newly loaded H3.3 is not turning over rapidly. Thus transplanted nuclei incorporate H3.3 readily from the oocyte, thereby changing their chromatin landscape and possibly reprogramming transcription.

### Timing of H3.3 incorporation and transcription

**Figure 2:**
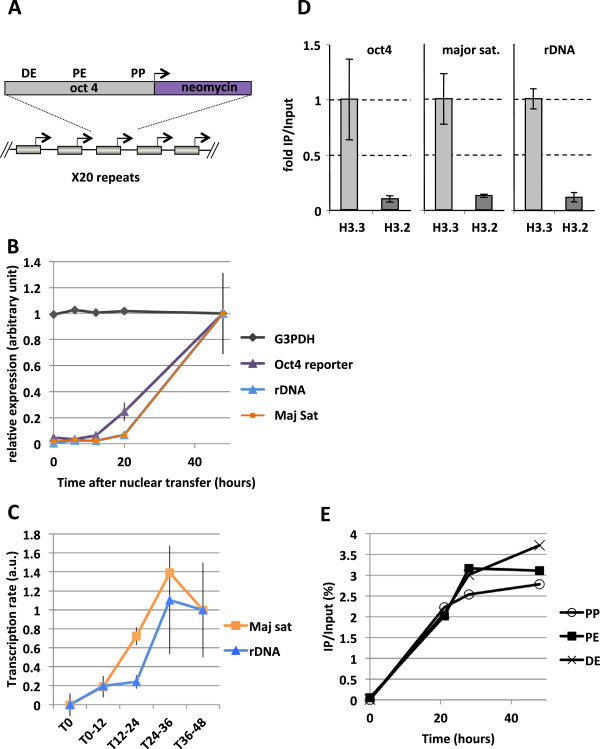
**Kinetics of transcription and H3.3 incorporation following nuclear transplantation to *Xenopus *oocytes.** (**A**) Schematic representation of the Oct4 transgene composed of a 20 copy array in the embryonic stem cells used in this study (ESC #5). DE, Distal enhancer; PE, Proximal enhancer; PP, Proximal promoter. (**B**) qRT-PCR analysis of transcription during a 48-h period following nuclear transplantation of 4 day-retinoic acid differentiated ESC #5 nuclei. Error bars indicate s.e.m. (*n* = 3). (**C**) Analysis of rDNA and major satellite transcription rates. Oocytes were transplanted with RA-ESCs and injected with BrUTP at various times after nuclear transfer (at 0, 12, 24, or 36 h). Oocytes were then collected 12 h after the time of BrUTP injection. In that way, the different samples collected will contain transcripts produced by the transplanted nuclei and labeled by BrUTP during a 12-h period (T12 to 24 h, T24 to 36 h, T36 to 48 h). After mRNA extraction BrUTP labeled transcripts are purified by immunoprecipitation with an anti-BrdUTP antibody and analyzed by qRT-PCR. Error bars indicate s.e.m. (*n* = 3). (**D**) ChIP analysis of histone incorporation 24 h after NT in oocytes preinjected with mRNA for HA-H3.3 and HA-H3.2, performed over the Oct4 reporter promoter, major satellite and ribosomal DNA genes, respectively. The total amount of incorporated H3.3 is set to 1. Error bars indicate standard deviation (*n* = 3). (**E**) Analysis of histone incorporation kinetics over Oct4 regulatory regions after NT. Four-day retinoic acid treated ESC #5 were transplanted in oocytes preinjected with mRNA for HA-H3.3 and HA-H3.2, collected over a 48-h time course and analyzed by ChIP with an HA antibody.

We have investigated how the association of histone H3.3 with transplanted nuclei relates to transcriptional reprogramming. Work in cultured cells has shown that H3.3 is present in specific parts of the genome. It is enriched in heterochromatic regions such as telomeres [6] and satellite repeats found at pericentric chromatin [15], as well as on ribosomal genes [16]. Lastly, H3.3 is accumulated on transcription factor binding sites in the regulatory regions of most genes as well as on the coding regions of transcribed genes in cultured mouse ES cells [6], and in cultured human HeLa cells [17]. We have therefore focused our analysis of H3.3 binding following nuclear transfer specifically to parts of the genome that reflect genomic H3.3 distribution in the cultured cells. We have thus monitored rDNA 28S genes (100 copies per genome) and major satellite repeats (approximately 3% of the mouse genome) [18]. Additionally, we have established a mouse embryonic cell line containing an array of approximately 20 copies of the 3.6 kB regulatory region of the Oct4 promoter fused to the coding region of neomycin resistance as reporter, here called ESC #5 (Figure 2A). Using that array we can monitor, by ChIP analysis, histone variant binding to a reporter gene. Induction of differentiation by retinoic acid (RA) treatment of ES cells is known to repress pluripotency gene transcription and to specifically invoke epigenetic changes resembling those of silent heterochromatin over the Oct4 gene [19], and we have taken advantage of this system to analyze silent chromatin reactivation after NT.

By qRT-PCR we have confirmed that 4-day retinoic acid (RA) treatment of the reporter ES cell line ESC#5 (RA-ESC#5) induces silencing of the endogenous pluripotency genes Sox2 and Oct4 as well as of the Oct4 transgene (Additional file 1: Figure S3). To confirm that the oocyte successfully reactivates the Oct4 transgene, a transcription analysis was carried out with RA-ESC #5 nuclei carrying the silent Oct4 array analyzed at various times after NT. We observe transcription of the rDNA 28S, major satellite repeat, as well as the previously silent Oct4 reporter within 12 to 20 h after nuclear transfer (Figure 2B). The level of all three kinds of transcript is greatly increased from 20 h to 48 h after transplantation. In particular, we note that transcripts from the rDNA and major satellite regions are accumulated to levels that largely exceed those observed in the donor nuclei immediately after nuclear transfer (at least 20-fold, Figure 2B, compare 0 h and 48 h after transplantation). The observed increase in rRNA and major satellite transcripts after nuclear transfer suggests transcriptional reprogramming of these parts of the genome too. However since both rDNA and pericentric heterochromatin can be transcribed in cultured cells, the observed accumulation of transcripts from these regions following nuclear transfer (Figure 2B) could result from a somatic type of transcription associated with increased transcript stability in the oocyte.

To confirm transcriptional reprogramming of these genomic regions we have directly measured whether their rate of transcription from transplanted nuclei changes after nuclear transfer. In order to measure changes in rate of transcription we have labeled new transcripts produced from transplanted nuclei by injecting BrUTP into the oocyte (Figure 2C). BrUTP was injected at different times after nuclear transfer and oocytes collected 12 h after injection. In that way the transcripts are labeled with BrUTP for 12-h periods covering the 48 h after nuclear transplantation. The labeled transcripts are then immunoprecipitated with an anti-BrdUTP antibody and analyzed by qRT-PCR, providing a measurement of transcript production per 12-h period. We observe that the rate of transcription from rDNA and major satellites increases approximately five times between the 12 to 24 h and 24 to 36 h periods that follow nuclear transplantation (Figure 2C). Therefore the reprogramming of transplanted nuclei to an oocyte type of transcription includes reactivation of silent genes such as Oct4, and an increased transcription of rDNA and pericentric chromatin. We point out that transcription of the latter has been observed in several instances, especially at the time of early embryonic development when heterochromatin is established [15,20].

We then compared the extent of the oocyte-expressed H3.3 and H3.2 incorporation into the transplanted chromatin analyzing different regions of the transplanted nuclei. For this purpose we expressed in the oocyte HA tagged H3.3 or H3.2 prior to nuclear transfer. The level of HA-H3 and HA-H3.3 obtained by mRNA injection is similar to that of their endogenous counterpart stored in the oocyte (Additional file 1: Figure S1 and Figure 1A). At 24 h after nuclear transfer of RA-ESC #5, the oocytes were cross-linked and analyzed by ChIP with an antibody recognizing the HA-tag in order to determine the extent of incorporation of histones from the oocyte. We found that HA-H3.3 was incorporated with an approximately 8-10-fold higher efficiency compared to the background level of HA-H3.2, not only over the Oct4 promoter but also over the major satellite region and ribosomal DNA (Figure 2D). Therefore the incorporation of oocyte H3.3 as opposed to H3.2 into transplanted nuclear chromatin as seen by immunofluorescence analysis (Figure 1C) is also observed by ChIP analysis. We conclude that H3.3 but not H3.2 is preferentially deposited on transplanted nuclear chromatin including regulatory regions such as in the Oct4 promoter.

Our results so far indicate that H3.3 deposition is an early event following NT but do not show whether it precedes or follows the switch from somatic type to oocyte type transcription. To address this we focused on Oct4 reactivation and investigated the time at which various parts of the Oct4 regulatory region exhibit histone H3.3 incorporation. The oocytes were injected with mRNA encoding HA-H3.3 as before and the next day RA ESC #5 nuclei were used for NT. The oocytes were then incubated and cross-linked at indicated time points prior to ChIP analysis for the well conserved proximal promoter (PP), proximal enhancer (PE), and the distal enhancer (DE) of Oct4. We found that histone H3.3 incorporation increased greatly during the first 20 h and then reached a plateau around 24 h after NT (Figure 2E). No difference in the extent of H3.3 incorporation between the promoter and enhancers was seen for up to 72 h, indicating a uniform exchange over the entire regulatory region of Oct4 after NT. At 20 h after nuclear transfer, both gene reactivation (qRT-PCR, Figure 2B) and H3.3 incorporation can be detected (ChIP, Figure 2E). However, whereas H3.3 incorporation on Oct4 regulatory regions reaches a maximum level around 24 h after nuclear transfer (Figure 2E), the quantity of accumulated Oct4 transcript increases massively after that time (Figure 2B). This observation is consistent with the hypothesis whereby H3.3 is incorporated onto a gene (and reach a maximum plateau level) while enabling activation and subsequent continuous accumulation of the transcribed mRNAs.

### H3.3 incorporation requires HIRA

**Figure 3:**
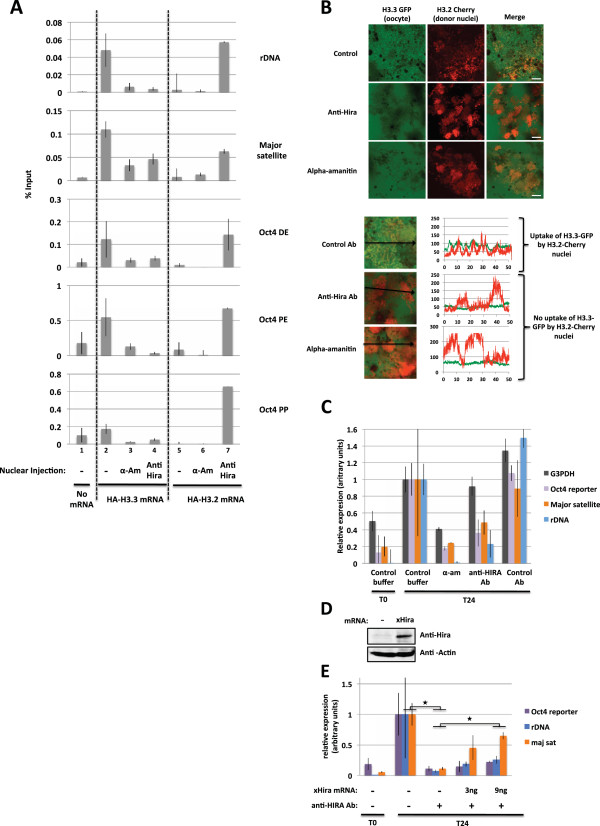
**A HIRA- and transcription- dependent H3.3 incorporation onto transplanted chromatin is required for transcriptional reprogramming following nuclear transfer.** (**A**) ChIP analysis of H3.2 and H3.3 incorporation onto transplanted chromatin following nuclear transfer to oocytes under conditions where transcription or HIRA is inhibited. Recipient oocytes were preinjected with either no mRNA, HA-H3.2 mRNA, or HA-H3.3 mRNA. RA-ESC #5 nuclei were then injected in the GV with or without co-injection of either alpha amanitin (RNA Polymerase II inhibition) or anti-HIRA Antibody (HIRA inhibition). Twenty-four hours after transplantation H3.2 and H3.3 incorporation onto various genomic regions was measured by ChIP analysis. Error bars indicate s.e.m. (*n* = 3). (**B**) Confocal analysis of H3.3 accumulation onto transplanted chromatin following nuclear transfer to oocytes in conditions where transcription or HIRA is inhibited. Oocytes were preinjected with H3.3-GFP mRNA. H3.2-cherry expressing ESC #5 nuclei were then transplanted to the oocyte in the presence of either alpha amanitin (RNA Polymerase II inhibition), anti-HIRA Antibody (HIRA inhibition), or control Antibody. Forty-eight hours after transplantation, H3.3-GFP loading onto chromatin was analyzed by confocal imaging of isolated GVs. Graphs show fluorescence intensity of donor H3.2-cherry (red) and oocyte H3.3-GFP (green) across a section of the image (black arrow). (**C**) qRT-PCR analysis of transcription 24 h following nuclear transfer of RA-ESC #5 nuclei in the presence of either alpha amanitin (RNA Polymerase II inhibition), anti-HIRA Antibody (HIRA inhibition), or control Antibody. Error bars indicate s.e.m. (*n* = 3). (**D**) Western blot analysis of *xenopus* HIRA expression 48 h following injection of HIRA mRNA to the oocytes. (**E**) qRT-PCR analysis of transcription 24 h following the transfer of RA-ESC #5 nuclei in the presence or absence of anti-HIRA antibody in GVs of oocytes pre-injected with various doses of HIRA mRNA. Error bars indicate s.e.m. (*n* = 3). ★ indicate *P* value < 0.05, (T-TEST).

We have next investigated whether H3.3 deposition on transplanted nuclei is a pre-requirement or a consequence of transcriptional reprogramming. We have first asked whether HIRA, known to be required for H3.3 incorporation into the paternal genome after fertilization [11] is also required for H3.3 deposition on nuclei following transplantation into the *Xenopus* oocyte. Recent studies describe a HIRA dependency for genome-wide H3.3 enrichment over transcribed regions of active genes in mouse ES or human HeLa cells [6,17]. However this incorporation of H3.3 seems not to be required for the maintenance of ongoing transcription in the ESCs [6,17]. HIRA dependent H3.3 deposition in transplanted nuclei was analyzed using a protein knockdown approach previously described and based on the co-injection of polyclonal antibodies together with the donor nuclei [5,21]. We used a polyclonal antibody that recognizes *Xenopus* HIRA and that has been previously used to immunodeplete HIRA protein from egg extract [9]. We verified that this antibody, when injected to the oocyte, was efficiently affecting DNA synthesis-independent chromatin assembly on plasmid DNA (Additional file 1: Figure S4). Oocytes expressing either HA-H3.3 or HA-H3.2 were then used as recipients for nuclear transfer. Retinoic acid-differentiated reporter ES cell nuclei were transplanted into oocytes, with the co-injection of an anti-HIRA antibody (to inhibit HIRA) or alpha-amanitin (to inhibit transcription). Twenty-four hours after nuclear transfer the oocytes were analyzed by ChIP and qRT-PCR. We observed that upon anti HIRA antibody injection, the deposition of maternally expressed HA-H3.3 on the Oct4 regulatory regions, rDNA, and major satellite repeats of the transplanted nuclei is reduced (Figure 3A, compare lane 2 with lane 4). This indicates that HIRA promotes histone H3.3 incorporation over the genomic region that we have analyzed in the transplanted nuclei.

Interestingly when HIRA mediated H3.3 deposition is inhibited an increased deposition of H3.2 is observed (Figure 3A, compare lane 5 with lane 7). In the presence of alpha-amanitin, neither H3.3 nor H3.2 is deposited onto transplanted chromatin (Figure 3A). We conclude that following nuclear transfer, H3.3 deposition on the tested genomic region is a HIRA- and transcription-dependent process. We have conducted a complementary analysis of H3.3 loading, this time on a global level, by monitoring H3.3-GFP accumulation onto transplanted chromatin by confocal microscopy. Similar to the ChIP analysis, we observed that H3.3 deposition is inhibited by both anti-HIRA antibody and alpha-amanitin injection (Figure 3B).

Together these data demonstrate that H3.3 deposition on a global nuclear level is HIRA- and largely transcription-dependent.

The alternative deposition of H3.2 in response to anti-HIRA injection is also observed on a global nuclear level when monitored by confocal microscopy, albeit on a subset of transplanted nuclei only (Additional file 1: Figure S5). This heterogeneous behavior of transplanted nuclei might reflect the various phases of the cell cycle that are represented in the donor nuclei population. Further investigations will be needed to describe the mechanism by which HIRA inhibition following nuclear transfer to *Xenopus* oocytes leads to H3.2 incorporation.

### H3.3 incorporation and transcription are interdependent

We next ask whether HIRA mediated H3.3 deposition is required for transcriptional reprogramming. Inhibition of HIRA by antibody injection greatly impairs transcriptional reprogramming as shown by the reduced expression of Oct4 reporter, rDNA, and major satellite from transplanted nuclei (qRT-PCR analysis, Figure 3C). Overexpression of HIRA by mRNA injection into oocytes prior to anti-HIRA antibody injection and nuclear transfer partially rescues the effect of antibody injection (Figure 3D and E). This observation confirms that the antibody effect is through inhibition of HIRA. We conclude that HIRA is required for the reprogramming from a somatic to an oocyte type of transcription. The alternative H3.2 deposition observed when HIRA is inhibited (Figure 3A) is not sufficient to promote transcriptional reprogramming (Figure 3C). This indicates that a specific deposition of H3.3 is required for efficient reprogramming.

## Conclusion

From a mechanistic point of view the requirement for H3.3 is mediated by HIRA. H3.3 incorporation and transcription seem to act in a cooperative and interdependent way. In cultured cells, inhibition of HIRA does not interfere with transcription [6]. However in the context of the developing embryo, HIRA activity is necessary at gastrulation where cell lineages are determined [12]. Together with these findings, our results reveal an involvement of this histone chaperone in the genome wide remodeling of transcription associated with major transition between cell states. Importantly, physical interactions between HIRA and the transcription machinery has recently been observed [17]. A requirement for H3.3 deposition in the reprogramming of somatic nuclei to an oocyte type of transcription may depend on an interaction between RNA polymerase II and HIRA.

## Methods

### Nuclear transfer procedure

Nuclei preparation, live confocal imaging, and ChIP analysis of transplanted nuclei have been previously described [5,22,23].

### TAU gel analysis

We prepared germinal vesicles (GV) soluble extracts [24], and soluble fractions from eggs and stage 28 embryos using the High Speed Egg (HSE) extract protocol [25]. We analyzed those extracts by electrophoresis in a Triton Acetic acid Urea (TAU) gel that separates histone subtypes [26].

### Antibodies

We used the following primary antibodies: anti-H3 (Abcam ab1791; 1:1,000 dilution), anti- H3.3 (Abnova H00003021-M01; 1:40), anti-HA (Roche Diagnostics Clone 3 F10; 1:1,000), anti-xHIRA ([9]; 1:2,000), anti-GFP (Santa Cruz #SC8334; 1:500), anti-actin (SIGMA; AC15, 1:5,000).

## Competing interests

The authors declare that they have no conflict of interest.

## Authors' contributions

JJ and JBG conceived and designed the experiments. JJ, CA, ES, NG, and JBG performed the experiments. JJ and CA analyzed data. GA contributed reagents/materials. JJ and JBG wrote the paper. CA, ES, and GA assisted in the preparation of the manuscript. All authors read and approved the final manuscript.

## Supplementary Material

Additional file 1 **Figure S1.** Level of GFP and HA tagged H3.2 or H3.3 expressed in *Xenopus* oocyte following mRNA injection. A total of 2 ng of mRNAs encoding H3.2-HA or H3.3-HA, or 3 ng of H3.2-GFP or H3.3-GFP mRNAs were injected to the cytoplasm of *Xenopus* oocyte. Forty-eight hours after injection proteins were extracted from the oocyte and subjected to WB analysis. Membranes were probed with antibodies specific for H3.3, HA, or GFP tags. Memcode staining served as a loading control. **Figure S2.** Kinetics of H3.3 deposition into transplanted nuclei. A time-lapse analysis was performed on H3.2-cherry expressing ESC nuclei transplanted into H3.3 GFP expressing oocyte germinal vesicles. Twenty-four hours after H3.3-GFP mRNA injection to the oocyte cytoplasm, the oocyte germinal vesicle (GV) was isolated in oil. H3.2-cherry ESC nuclei were then transplanted to these oil-GVs and analyzed by confocal microscopy for a period of 15 h. **Figure S3.** qRT-PCR analysis of gene expression in reporter ESCs with or without retinoic acid differentiation. ESCs containing an Oct4 reporter **(**ESC #5) were cultured either in the presence of LIF or in the absence of LIF and with retinoic acid (1 μM) for a period of 5 days. Cells were then collected, mRNA extracted, and gene expression was measured by qRT-PCR. **Figure S4.** DNA synthesis independent chromatin assembly on plasmid DNA injected to oocyte is inhibited by anti-HIRA antibody. *In-vivo* chromatin assembly assay. We performed *in-vivo* chromatin assembly according to Roche *et al.*, *Methods Mol Biol*., 129–47, 2006. Circular dsDNA plasmid pBS0 (10 ng) was injected to *Xenopus* oocyte germinal vesicle together with 16 nL of water (-), preimmune serum, or anti-HIRA antibody solutions. DNA synthesis independent chromatin assembly was then allowed to proceed by incubating the oocyte for different amount of time (from 15 to 180 min), after which plasmid DNA was recovered, deproteinized, and analyzed by electrophoresis. The presence of the anti-HIRA antibody inhibits chromatin assembly on the injected plasmid, as shown by reduced supercoiling. **Figure S5.** Confocal analysis of H3.3 or H3.2 accumulation onto transplanted chromatin following nuclear transfer to oocytes in conditions where HIRA is inhibited. Oocytes were preinjected with H3.3-GFP or H3.2-GFP mRNA. H3.2-cherry expressing ESC #5 nuclei were then transplanted to the oocyte in the presence of anti-HIRA Antibody or control Antibody. Forty-eight hours after transplantation, H3.3-GFP (top panel) or H3.2-GFP (bottom panel) loading onto chromatin was analyzed by confocal imaging of isolated GVs. Images are Z-stacks projection. Click here for file
