# Supplementary figures and images for: HIRA dependent H3.3 deposition is required for transcriptional reprogramming following nuclear transfer to Xenopus oocytes
Source: Epigenetics Chromatin. 2012 Oct 29;5:17. doi: 10.1186/1756-8935-5-17 (PMC3538669; doi:10.1186/1756-8935-5-17)

S2

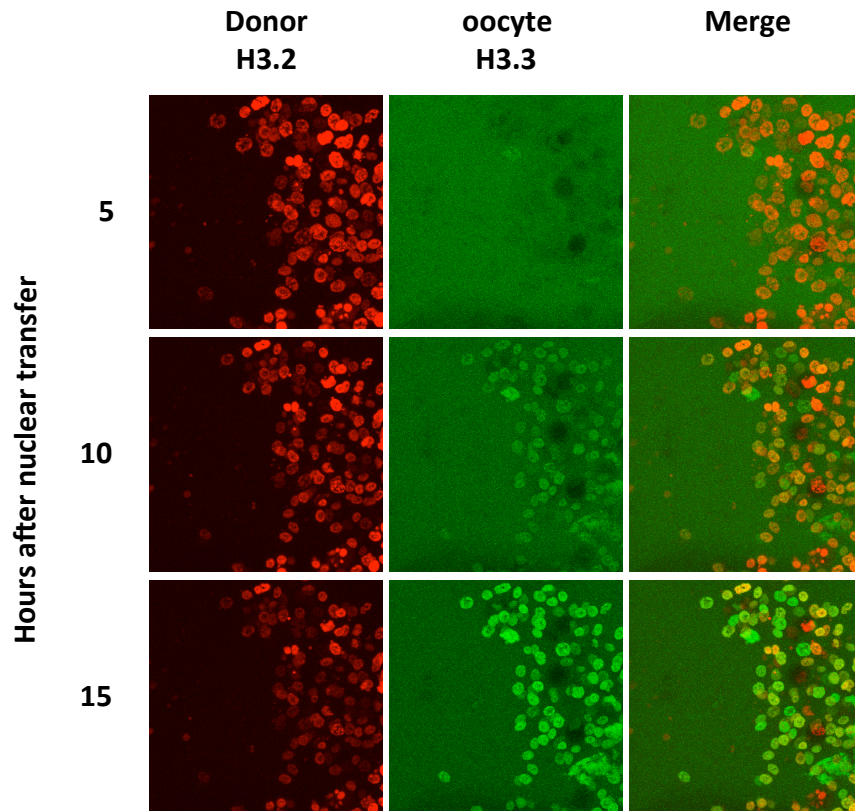

S3

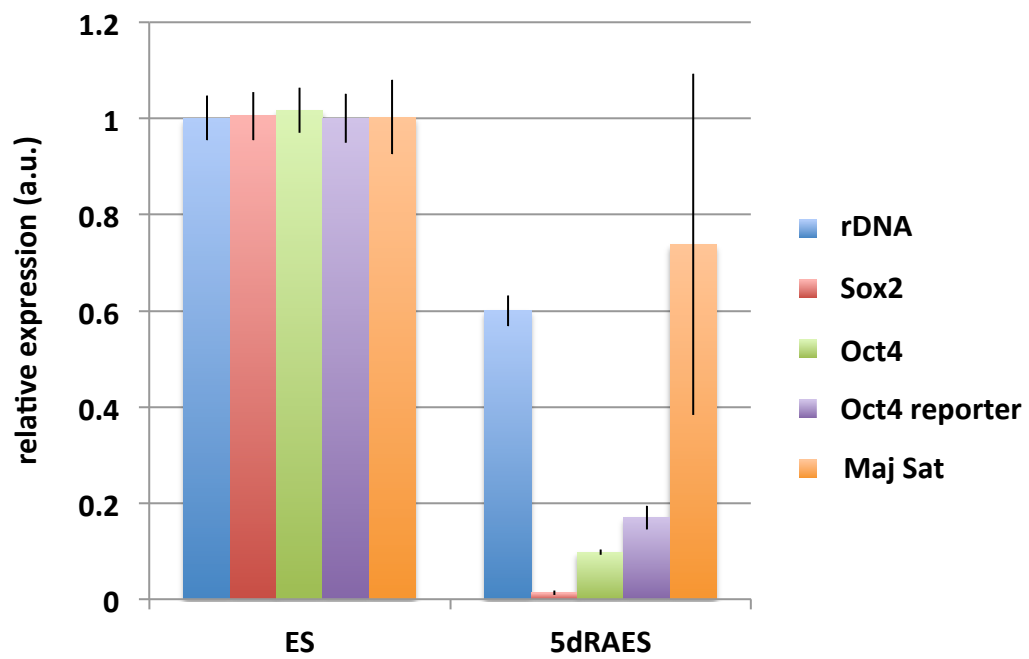

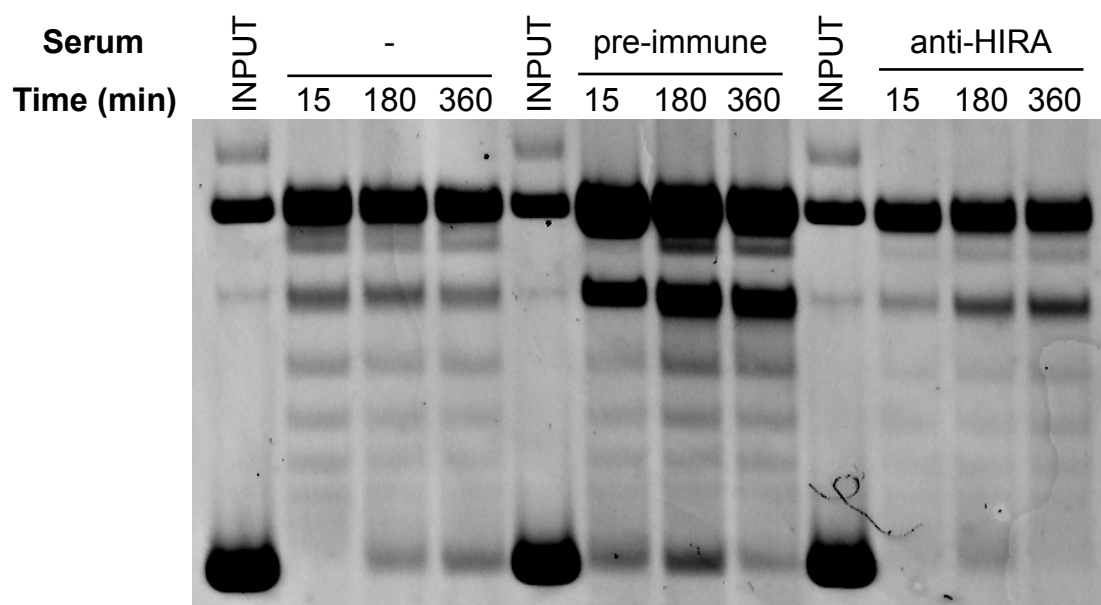

S5

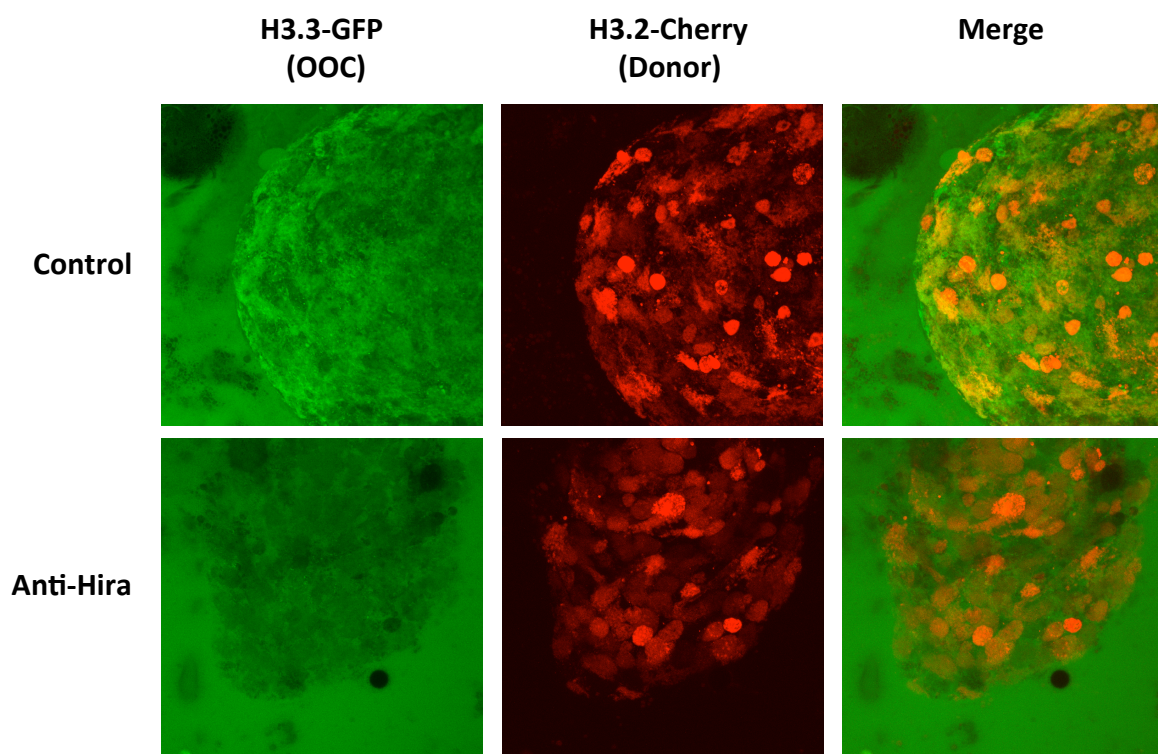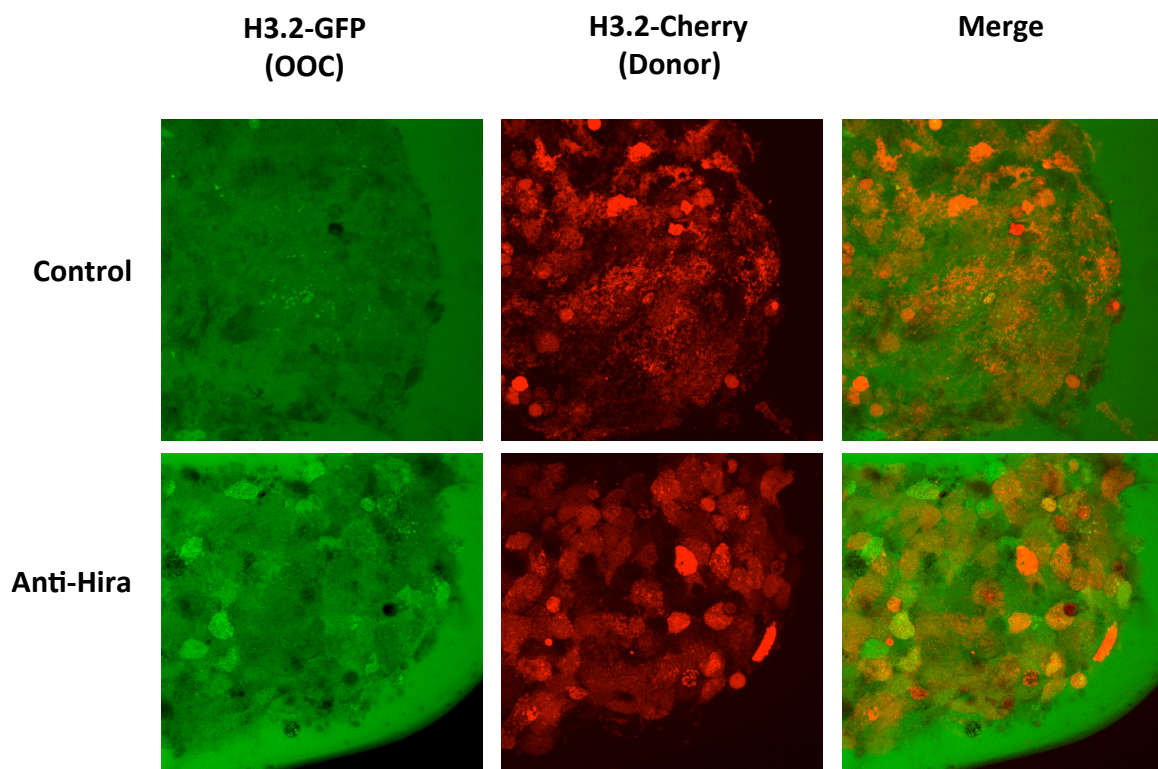

Supplement: Additional file 1 — Figure S1. Level of GFP and HA tagged H3.2 or H3.3 expressed in Xenopus oocyte following mRNA injection. A total of 2 ng of mRNAs encoding H3.2-HA or H3.3-HA, or 3 ng of H3.2-GFP or H3.3-GFP mRNAs were injected to the cytoplasm of Xenopus oocyte. Forty-eight hours after injection proteins were extracted from the oocyte and subjected to WB analysis. Membranes were probed with antibodies specific for H3.3, HA, or GFP tags. Memcode staining served as a loading control. Figure S2. Kinetics of H3.3 deposition into transplanted nuclei. A time-lapse analysis was performed on H3.2-cherry expressing ESC nuclei transplanted into H3.3 GFP expressing oocyte germinal vesicles. Twenty-four hours after H3.3-GFP mRNA injection to the oocyte cytoplasm, the oocyte germinal vesicle (GV) was isolated in oil. H3.2-cherry ESC nuclei were then transplanted to these oil-GVs and analyzed by confocal microscopy for a period of 15 h. Figure S3. qRT-PCR analysis of gene expression in reporter ESCs with or without retinoic acid differentiation. ESCs containing an Oct4 reporter (ESC #5) were cultured either in the presence of LIF or in the absence of LIF and with retinoic acid (1 μM) for a period of 5 days. Cells were then collected, mRNA extracted, and gene expression was measured by qRT-PCR. Figure S4. DNA synthesis independent chromatin assembly on plasmid DNA injected to oocyte is inhibited by anti-HIRA antibody. In-vivo chromatin assembly assay. We performed in-vivo chromatin assembly according to Roche et al., Methods Mol Biol., 129–47, 2006. Circular dsDNA plasmid pBS0 (10 ng) was injected to Xenopus oocyte germinal vesicle together with 16 nL of water (-), preimmune serum, or anti-HIRA antibody solutions. DNA synthesis independent chromatin assembly was then allowed to proceed by incubating the oocyte for different amount of time (from 15 to 180 min), after which plasmid DNA was recovered, deproteinized, and analyzed by electrophoresis. The presence of the anti-HIRA antibody inhibits chr [file 1756-8935-5-17-S1.pdf]
